# Advancing mold identification in the routine laboratory, performance of smartphone-based imaging and a newly developed convolutional neural network

**DOI:** 10.1128/spectrum.02924-25

**Published:** 2025-12-16

**Authors:** Lukas Weber, Sarah Brueningk, Bettina Schulthess, Gloria Stillhart, Michelle Bressan, Nadja Pulver-Vontobel, Jasmin Schimetzki, Rosmi Puthan, Andrea Egli-Berini, Oliver Nolte, Adrian Egli

**Affiliations:** 1 Institute of Medical Microbiology, University of Zurich 27217, Zurich, Switzerland; 2 Department of Radiation Oncology, Inselspital, Bern University Hospital and University of Bern 571565, Bern, Switzerland; 3 Bildungszentrum Gesundheit 128918 , Basel-Stadt, Switzerland; Houston Methodist Hospital, Houston, Texas, USA

**Keywords:** diagnostic mycology, machine learning, mycology, artificial intelligence, deep learning

## Abstract

Mold identification in clinical diagnostics is traditionally labor-intensive and is dependent on expert interpretation. MoldVision is a deep-learning approach that uses smartphone images of mold cultures to automate identification. We analyzed 161 clinical isolates across four common mold genera: *Penicillium* spp., *Aspergillus* spp. (with *A. flavus* and *A. fumigatus*), *Fusarium* spp., and *Cladosporium* spp. Daily images were captured from the top and bottom of culture plates over 5 days using a standardized smartphone setup, generating over 4,000 images. We trained three variations of Visual Geometry Group 16 (VGG16) convolutional neural networks (CNNs) and benchmarked the best-performing model (VGG16 with dual classification heads) against LightGBM models trained on pre-extracted features and human expert assessments at various time points. The best-performing VGG16 model achieved a mean (SD) AUROC of 92.7% ± 1.8% and sensitivity of 68.7% ± 2.6% across all species. Here, the performance in identifying *Cladosporium* spp. was best (AUROC of 99.9% ± 0.1%, fivefold cross-validation mean and SD). Regarding the evaluations over time, early-stage classification (days 1–2) was challenging (F1-score 38.8% ± 3.5% across all species) but improved significantly on days 3–5 (F1 92.1% ± 2.8% across all species). Compared to experts, MoldVision consistently showed superior performance, particularly in mature cultures, detecting subtle morphological features earlier and more accurately. Our results demonstrate that CNNs integrated with low-cost smartphone imaging can reliably classify mold species in routine diagnostics, outperforming human experts in many cases. This approach offers a practical and scalable solution for laboratories lacking specialized mycology expertise, especially in resource-limited settings.

**IMPORTANCE:**

Timely, accurate mold identification can save lives, yet today it often requires days of growth and scarce expert time. MoldVision offers a practical alternative: use a standard smartphone to photograph routine agar plates and let an AI model aid with species recognition. Trained on thousands of images across 5 days of growth, the system detects tell-tale colony patterns as they appear and, once growth is established (days 3–5), often matches or exceeds human readers. This could shorten reporting, prioritize which cultures need expert review, and extend reliable mycology support to laboratories without specialized equipment or staffing. Because it relies on affordable tools and a standardized imaging workflow, MoldVision is scalable to resource-limited settings. We also share data and code to encourage external validation and improvement, moving this concept toward faster and equitable fungal diagnostics. These findings highlight a practical path to amplify diagnostic capacity where it is needed most.

## INTRODUCTION

Invasive mold infections are rare (1) but are associated with devastating complications, primarily affecting at-risk populations such as immunocompromised hosts and intensive care patients (2). Mold infections show considerable morbidity and mortality (3). Prompt diagnosis is crucial for the initiation of adequate antifungal treatment. However, the current gold standard, culture-based identification by experts trained to characterize macroscopic and microscopic features of molds, is slow, typically requiring 3 to 7 days from sample collection to definitive identification (4). Furthermore, species-level identification using traditional microscopy demands significant expertise and long-term training, implying that required human resources are often unavailable outside specialized laboratories (5, 6).

Alternative diagnostic techniques, such as matrix-assisted laser desorption/ionization time-of-flight mass spectrometry (MALDI-TOF MS), offer more rapid, rater-independent identification but still rely on culture growth and are strongly dependent on the quality of the underlying database. As such, 3 to 4 days of laboratory observation are required before analysis can begin (7, 8). The specialized and expensive equipment needed for MALDI-TOF further restricts its availability, and existing databases often lack diverse fungal isolates (9). Species-specific nucleic acid amplification tests (NAATs) or internal transcribed spacer (ITS) sequencing-based methods have emerged, offering higher sensitivity and specificity compared to traditional methods, i.e., sensitivity up to 84.0% and specificity up to 76% were reported (10).

Nevertheless, NAAT and ITS sequencing still demand specialized infrastructure, skilled personnel, and prior knowledge of the genetic sequences of the target organism, limiting their utility for novel or poorly characterized species (11).

Despite the utility of computer vision for rapid image analysis, its deployment in routine mold diagnostics has been limited by the lack of comprehensive, publicly available data sets capturing the longitudinal growth of mold in standardized diagnostic settings, and by regulatory demands. In addition, the color and morphology of mold colonies can be strongly influenced by factors such as culture age and growth medium, complicating purely morphological identification methods (12).

Here, we propose an approach that leverages convolutional neural networks (CNNs) for the automated identification of mold colonies in routine laboratory settings from smartphone images (13). CNNs excel in medical imaging and diagnostics (14–18), learning hierarchical features, i.e., shape, texture, and color, from images without the need for extensive manual feature engineering. In this study, we introduce a CNN-based system, MoldVision, to automatically detect and classify mold colonies on agar plates. Our method uses cost-effective, commercial off-the-shelf tools—such as modern smartphones with high-quality cameras—to support widespread adoption. The concept of CNNs is described in greater detail in the supplementary text (Text S1, Fig. S1).

The goals of this study are (i) to develop and train CNN models on longitudinal images of mold cultures for rapid, accurate mold identification in routine laboratory settings, and (ii) to evaluate these predictions against visual inspections performed by experienced laboratory technicians.

## MATERIALS AND METHODS

**Fig 1:**
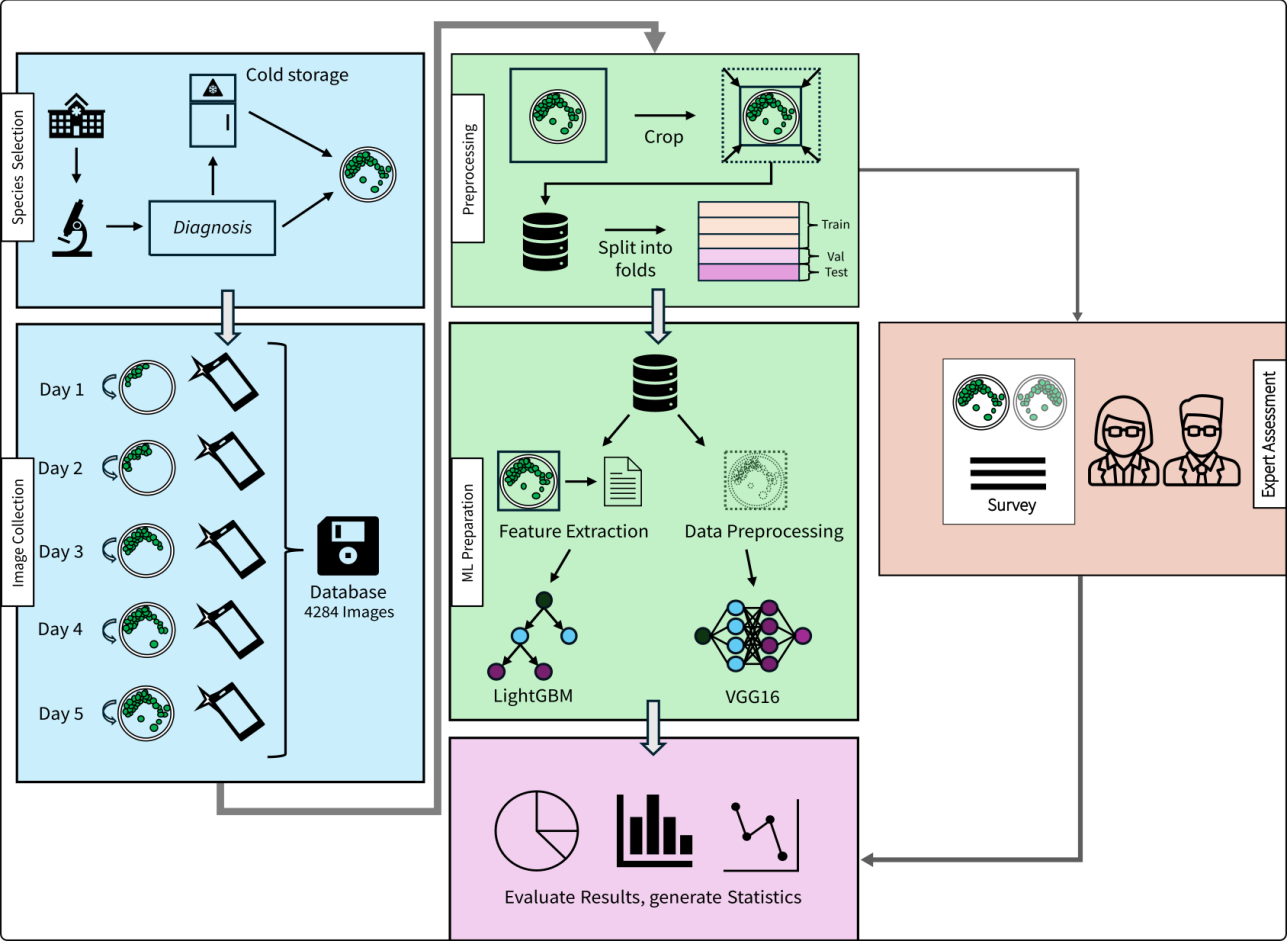
Methodological overview. Strains from the institute’s strain collection were used and cultured over 5 days on standardized agar plates under conditions according to internal standard operating procedures. Each day, an image was acquired from the top and bottom of the plate with a standard smartphone (OPPO A94 5G; blue part). The image set was split into training, validation, and test data sets to train different machine and deep-learning classifiers (green part). Output was explored using different metrics (pink part). The prediction performance was compared to expert human observers (red part).

The project was conducted at the routine diagnostics lab of the Institute of Medical Microbiology at the University of Zurich. An overview of the study workflow, including species collection, image acquisition, data preprocessing, model development, and evaluation, is presented in Fig. 1.

### Species selection and culture conditions

Five frequently isolated molds from clinical samples were selected from the strain collection of the Institute of Medical Microbiology at the University of Zurich: *Penicillium* spp., *Aspergillus flavus*, *Aspergillus fumigatus*, *Fusarium* spp., and *Cladosporium* spp. No obvious duplicates, e.g., the same strain isolates taken twice from the same patients, were included. All taxonomic classifications were confirmed by microscopy according to the diagnostic routine procedure and following internal quality management under the ISO norm 17025. Notably, *A. flavus* and *A. fumigatus* are closely related and exhibit similar morphological features, providing a challenging scenario for granular differentiation.

All strains were sub-cultured on Sabouraud Gentamicin and Chloramphenicol Agar plates (BD, Allschwil, Switzerland) and incubated for 5 days at 25°C in a humidified environment, generating colonies with distinct growth characteristics over the observation period. The culturing procedure followed was identical to that used in the ISO-accredited routine.

### Imaging data acquisition

A custom-made dark metal box with a black interior (Fig. S2) was used to minimize external light reflections during photography. For image acquisition, an OPPO A94 5G smartphone was selected, offering both affordability and suitable camera performance for the study’s requirements. The smartphone was placed above the agar plate for image capture. Each day, images were taken of both the top and the bottom of each agar plate at three different rotational angles (±30°), yielding six images per isolate per day. To ensure consistency, the camera settings were fixed at an aperture of f/1.7, exposure compensation of 0, focal length of 4.71 mm, and a shutter speed of 1/50 s. White balance was set to 5,500 K to maintain consistent color reproduction, and ISO sensitivity was dynamic but remained between 1,400 and 1,600. Over the 5 day period, each isolate was documented with up to 30 images. For technical and methodological reasons, it was not always possible to successfully capture a full spread of images each week.

Multiple lab personnel (*n* = 4) performed the photography following a standardized written protocol. Images were included if they met the following criteria: (i) visible growth of the target species, (ii) absence of cross-contamination, and (iii) suitable image quality based on subjective evaluation by study personnel. For each photograph, the following metadata were recorded: species label (true class), date and time of acquisition, a unique isolate identification number, and a plate identifier. All images were cropped to the bounding box of the agar plate using an automated script in OpenCV (19). OpenCV is an open-source computer vision and machine learning library that provides tools for image processing, feature detection, and camera calibration, widely used in academic and industrial applications.

### Data splitting and cross-validation

To account for variability and ensure robust estimates of model performance, a fivefold nested cross-validation strategy was employed. An 80-20 split was used in each outer and inner fold (train/validation; train/test), stratified by species (test set *n* ≈ 860 images, validation set *n* ≈ 680 images, training set *n* ≈ 2,750 images). Additionally, images were grouped by plate identifier (i.e., all images of the same plate were either within the train, test, or validation set) to avoid leakage of highly similar samples (i.e., images from the same plate) between training and test sets. As a cross-validation strategy was employed, the test set was created from the main data set but remained unseen by the model during training and validation. It was only used once, after model development and hyperparameter optimization were complete, to provide an unbiased estimate of the final model’s performance. Any performance is reported as mean values and standard deviations across test sets.

### Image classification

Image-based predictions are typically obtained by deep-learning models from the realm of CNNs. Given the considerable number of tunable parameters, these models, however, typically perform best if trained on substantial amounts of images.

Alternatively, standard machine learning models can also be trained on pre-extracted features. Here, we compare these two approaches, using a deep-learning architecture based on the Visual Geometry Group 16 (VGG16) model with feature extraction performed using processes provided by VGG16 (20) and Light Gradient Boosting Machine (LGBM) (21) on pre-extracted image features.

#### VGG16 pipeline

**Fig 2:**
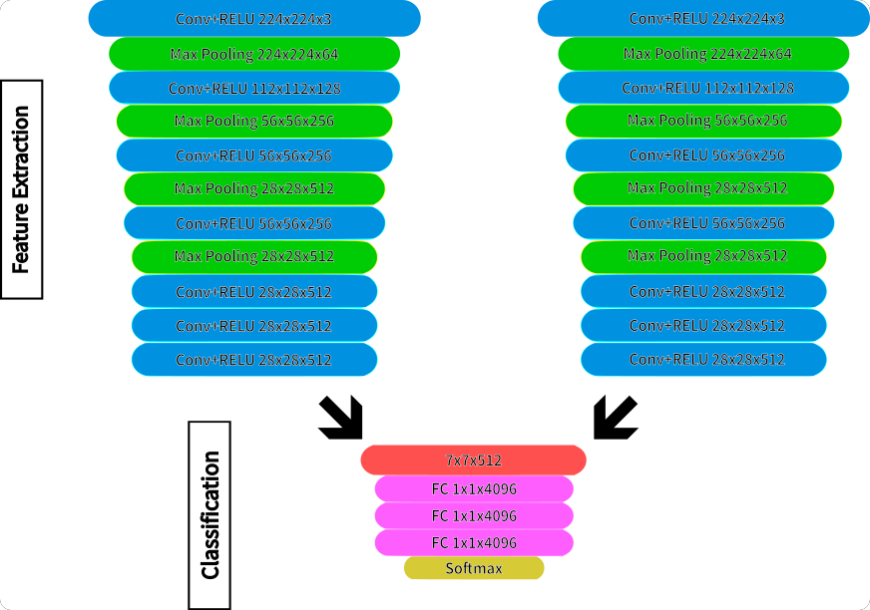
MoldVision deep-learning architecture. Building on the VGG16 design, this variation was adapted to account for paired processing of top and bottom images of the plates. Conv, convolutional; RELU, rectified linear unit; FC, fully connected layers. Not displayed are details of concatenation processes.

Images were preprocessed by re-sizing to 256 × 256 pixels using bilinear interpolation, which computes each new pixel value as a weighted average of the four nearest pixels to produce smoother transitions and reduce distortion. This was followed by a center crop to 244 × 244 pixels, in which the crop window was positioned equidistant from all image edges to retain the central region of interest. The final step was pixel value normalization, the process of adjusting image pixel intensities to a common scale by subtracting a mean value and dividing by a standard deviation, so that the input distribution matches what a model was trained on, using mean and standard deviation arrays recommended for VGG16 (22). Deriving from the VGG16 backbone (14, 23, 24), each image view (top, bottom) is passed into its own stream of convolutional layers (referred to from here as “dual-heading”), effectively replicating the well-established 16-layer VGG16 configuration (3 × 3 convolutions, 2 × 2 max pooling, Fig. 2). We implemented this using PyTorch 2.1.0 (25) with PyTorch Lightning 2.2.1 (26) as a framework and retained the original pre-trained weights for the initial convolutional blocks to preserve low-level edge/texture filters learned from large-scale natural images, leveraging transfer learning from ImageNet-scale image recognition. Higher-level blocks and all fully connected layer weights were trainable, allowing the network to adapt to fungal-specific textural features, e.g., sporulation patterns, colony rim definition, and reverse pigmentation.

Model training was conducted with PyTorch’s Stochastic Gradient Descent (momentum = 0.9) and Cross Entropy Loss. Early stopping (patience of five epochs) evaluated on the internal validation data fraction was used to account for model overfitting. We performed hyperparameter tuning by grid search of the learning rate (initialized at 0.05 and reduced by a factor of 0.1 after every 10–20 epochs if the validation loss plateaued) and batch size (set to 32, balancing computational efficiency and gradient stability). We refer to the most successful VGG16 dual-head model as “MoldVision.”

We further investigated two alternative architecture designs, an unmodified pretrained VGG-16 architecture, and VGG16_6Chan, in which the input layer was expanded from three to six channels to allow the model to ingest the top- and bottom-layer inputs simultaneously from the outset without the dual-head approach mentioned above. Full implementation details are provided in the Supplementary Section (Text S2). Neither of these architectures improved performance beyond the above-mentioned approach.

#### Standard machine learning baseline (LGBM)

Images were resized to 500 × 500 pixels, gray-scaled, normalized, and subjected to a circular mask to exclude regions outside the agar plate. Feature libraries (e.g., color histograms, local binary patterns, and average contour) were extracted using OpenCV 4.8.1 (19) and scikit-learn 1.3.2 (27). We trained a gradient-boosting model (LGBM) (21, 28, 29), implemented based on the lightgbm 4.0.0 Python library with grid search for hyperparameter optimization on internal validation subsets (Text S3). The model was trained for up to 2,000 iterations, with early stopping triggered once the validation loss did not improve for 100 consecutive steps as a robust baseline against the CNN-based approach.

### Expert observer rating

To benchmark the data-driven predictions against human expertise, a survey was conducted using Google Forms. A sample of the original data set was selected (*n* = 421), forming one top and one bottom image per day for each species out of the data set as a representative sample. Five laboratory technicians with experience in fungal diagnostics were asked to name the species (no “none” option) and indicate their subjective confidence level on a 1–10 scale (1 = “random guess,” 10 = “complete confidence”). They were further prompted to describe key features guiding their decision, e.g., color, shape, and growth characteristics.

### Performance assessment

The following metrics were calculated using scikit-learn implementation: accuracy, sensitivity, specificity, F1 score, area under the receiver operating characteristic curve (AUROC), and area under the precision-recall curve (AUPRC). Any reported values represent mean and standard deviation across the five test sets. We assessed performance across and within species as well as longitudinally over the 5 day culture period. The final reported species in routine diagnostics, based on the ISO-accredited workflow, served as the ground truth. This also includes a layer of microscopic identification. Notably, this study did not validate using isolates or images sourced from external laboratories.

## RESULTS

### Overall performance

A total of 161 individual fungal isolates were cultured, yielding 4,500 images. Of these, one isolate was removed due to total contamination of the sample, and 216 images (4.8%) were excluded due to lack of visible growth, cross-contamination, or poor image quality, while maintaining approximate class balance (Table 1). The following mold genera were included: *Penicillium* spp., *Aspergillus* spp. (including *A. flavus* and *A. fumigatus*), *Fusarium* spp*.*, and *Cladosporium* spp*.*

**TABLE 1:** Final image count and class prevalence per species^*a*^

Species	# Isolates (*n* = 160)	# Images (*n* = 4,284)	Class prevalence
*Penicillium* spp.	32	864	20.0%
*Aspergillus flavus*	32	852	20.0%
*Aspergillus fumigatus*	33	882	20.6%
*Fusarium* spp.	33	882	20.6%
*Cladosporium* spp.	30	804	18.8%

^*a*^ This table reports the number of isolates (total *n* = 160), the total images including obverse and reverse images collected per species (total *n* = 4,284), and the relative prevalence of each class (percentage of overall images) contributing to the final data set, for each of the five target species.

Across all metrics and all time points, MoldVision consistently outperformed a prediction based on pre-extracted image features or alternative architecture designs based on VGG16 (Table 2). Notably, MoldVision achieved a mean AUROC of 92.7 ± 1.8% and a mean AUPRC of 81.8 ± 3.1% across cross-validation folds and species, with a positive class prevalence of 20.0 ± 2.0%. All CNN-based models showed better performance overall than LGBM. Given these results, MoldVision was selected for more in-depth analysis.

**TABLE 2:** Comparison of machine learning models over all species (mean ± SD)^*a*^

Model	Recall^*b*^	Specificity	Precision	F1^*c*^	AUROC	AUPRC^*d*^
LGBM	46.5 ± 4.8%	46.5 ± 4.8%	47.1 ± 3.9%	46.4 ± 4.8%	76.6 ± 3.5%	52.3 ± 4.9%
VGG16	63.3 ± 1.0%	90.8 ± 0.2%	63.7 ± 0.8%	63.3 ± 1.0%	89.0 ± 0.3%	73.6 ± 0.9%
VGG16_6Chan	58.1 ± 2.9%	89.5 ± 0.7%	58.3 ± 2.7%	58.2 ± 2.9%	85.5 ± 2.2%	67.2 ± 4.0%
MoldVision	68.7 ± 2.6%	92.1 ± 0.7%	72.0 ± 2.0%	68.7 ± 2.6%	92.7 ± 1.8%	81.8 ± 3.1%

^*a*^ AUROC was the primary statistic and was higher for MoldVision than all other models. Median paired difference = 0.085 (95% CI: 0.062–0.109); Wilcoxon signed-rank V = 15, *P* = 0.031 (one-sided). Other metrics (F1, recall, precision, specificity, AUPRC) showed the same fold-wise direction with the same Wilcoxon *P* and positive median paired differences. Shaded rows is Moldvision, as it performed best in all metrics.

^*b*^ Identical to sensitivity; calculated for listed precision.

^*c*^ F1 score: harmonic mean of precision and recall.

^*d*^ Area under the precision–recall curve.

### Performance over time

**Fig 3:**
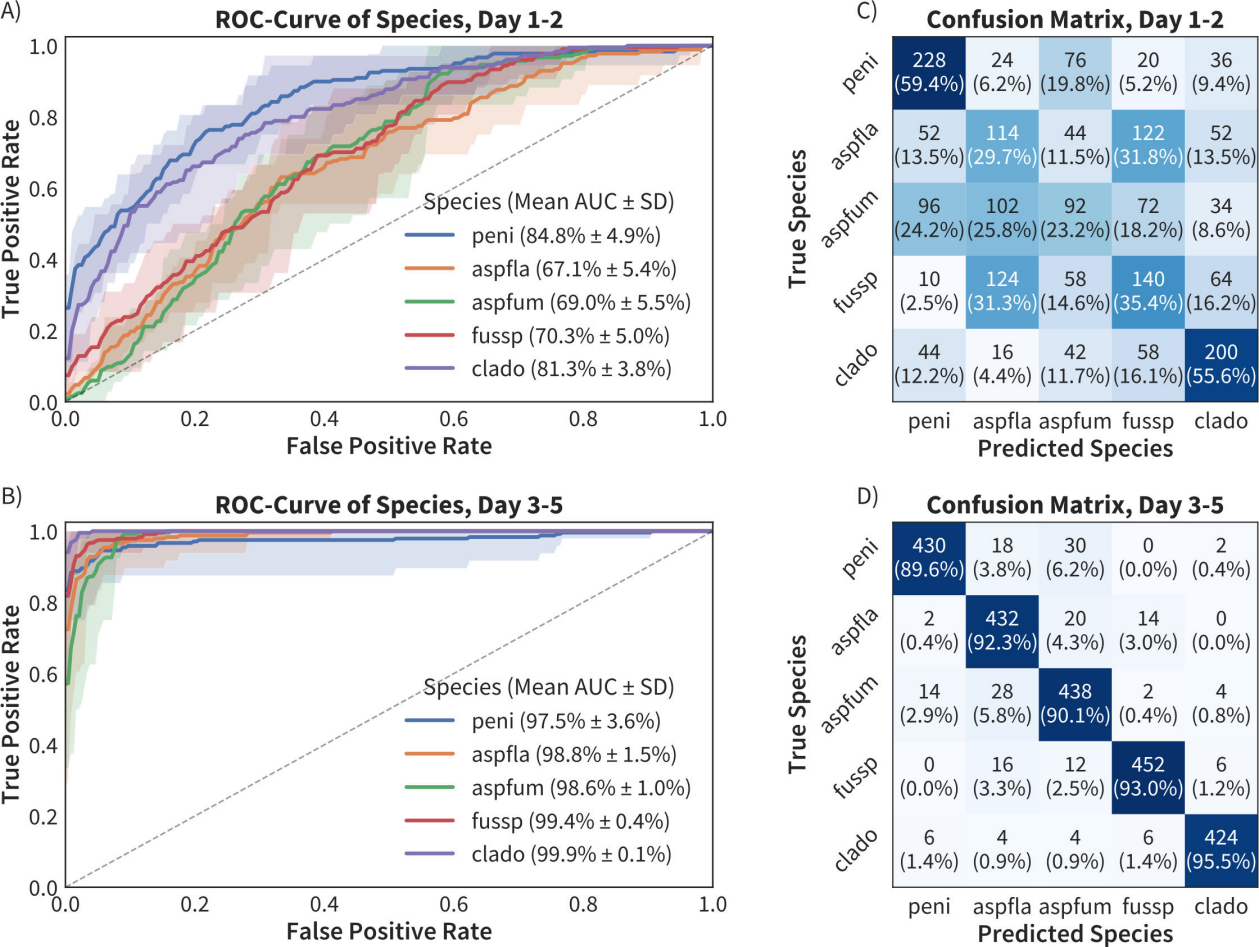
Receiver operating curves (ROC) and confusion matrices of species classification at early (days 1–2) and late (days 3–5) growth stages. ROC curves (left, **A and B**) and confusion matrices (right, **C and D**) depict the performance of species classification models for *peni, Penicillium* spp*.; aspfla, A. flavus*; *aspfum, A. fumigatus*; *fussp, Fusarium* spp*.*; and *clado, Cladosporium* spp. in the early and late stages. The curves of the ROC show a standard deviation represented by the transparent cloud.

To explore how colony maturation influenced classification, images were partitioned into early (days 1–2) and late (days 3–5) growth stages, and separate MoldVision models were trained on both data set splits. In the early stage, many colonies had only minimal or indistinct morphological and color features, leading to lower classification accuracy for *A. fumigatus* and *A. flavus*. These species yielded AUROC values of 67.1 ± 5.4% and 69.0 ± 5.5%, respectively (Fig. 3A), and were misclassified across multiple classes. In contrast, *Penicillium* spp*.* and *Cladosporium* spp. were better differentiated early on, with AUROC values of 84.8 ± 4.9% and 81.3 ± 3.8%, and confusion matrix concordance of 59.4% and 55.6%, respectively (Fig. 3C). From day 3 onward, colony characteristics became more distinct, and model performance improved markedly (Fig. 3B and D). The concordance between predicted and true species exceeded 89.6% for all species, with AUROC values surpassing 97.5%. This finding underscores the importance of sufficient colony growth time for reliable automated identification.

### Comparison to human expertise

**Fig 4:**
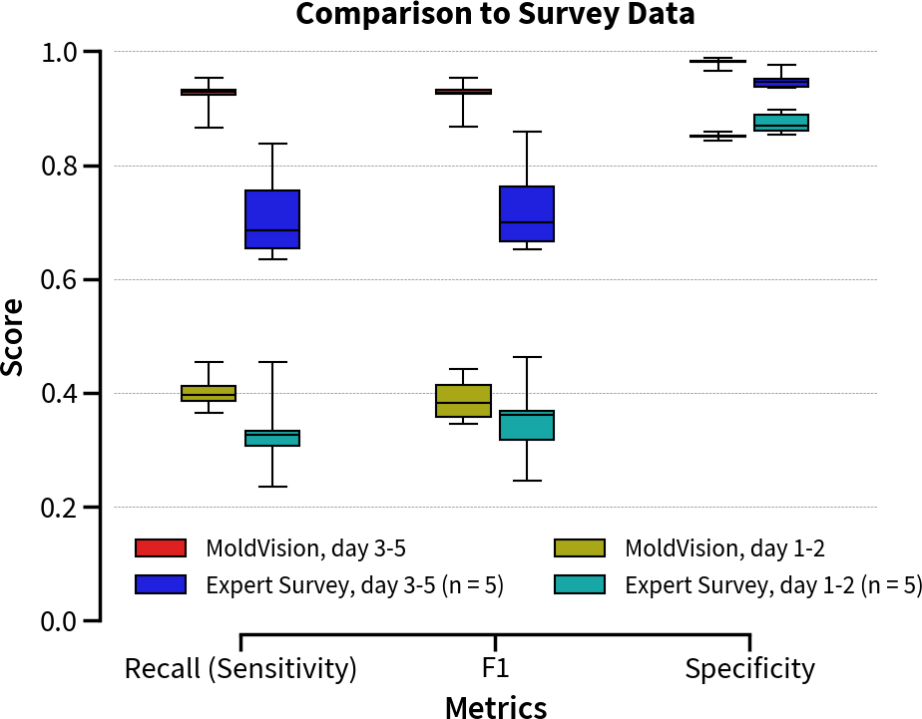
Comparison of MoldVision and human expertise for classification performance over aging cultures: Box plots display the performance comparison of MoldVision (days 1–2 and days 3–5) to human expert data (*n* = 5) in terms of recall (sensitivity), F1 score, and specificity. The MoldVision model in the late stage (red) shows superior performance across all metrics, particularly in specificity.

To further contrast the model’s classification accuracy with expert assessments, we conducted a survey with laboratory technicians (*n* = 5, four diagnostic lab technicians and one laboratory scientist candidate with between 3 and 20 years of experience). Participants provided identifications for both early (days 1–2) and late (days 3–5) colony images, using the same top- and bottom-view photographs presented to MoldVision. As summarized in Fig. 4, MoldVision achieved higher median values for recall, F1-score, and specificity in both early and late assessments compared to human survey data. Specifically, for late time points, MoldVision attained a median recall of ≈ 90% and specificity exceeding 95%, while human experts averaged lower recall (≈ 70%) and specificity (≈ 90%). These differences were even more pronounced in the F1 metric, where the model consistently outperformed the human group by a margin of 10%–20%.

Likewise, on days 1–2, both MoldVision and expert assessments showed lower overall scores, reflecting the limited morphological cues available. Nevertheless, MoldVision again demonstrated superior performance, with median recall and specificity 10%–15% higher than the expert survey results.

**Fig 5:**
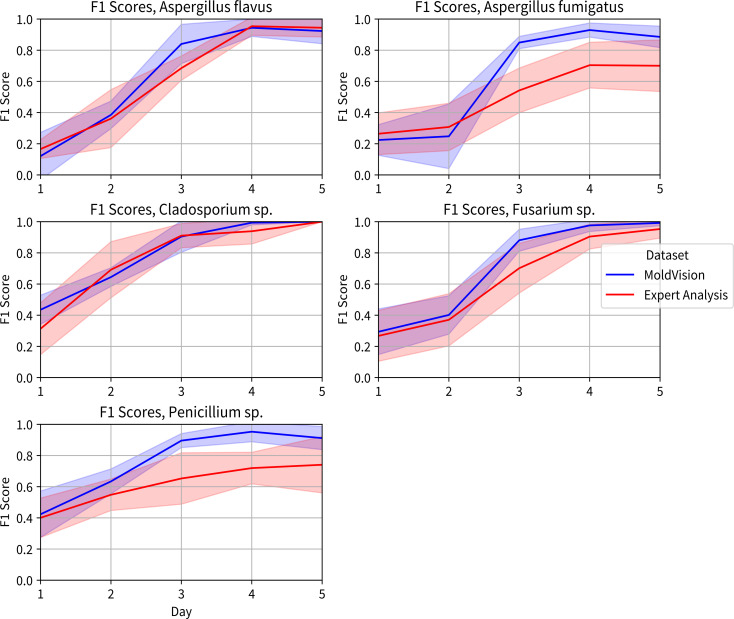
Temporal F1 score trajectories over a 5 day period, comparing MoldVision (blue) and human expertise (red): panels plot the mean F1 score (*y*-axis) against day of observation (*x*-axis, days 1–5) for a single species. Shaded envelopes around each curve indicate one standard deviation of performance across test replicates. By day 3, MoldVision (blue) begins to outperform expert analysis (red) in most species, with F1 scores converging toward 0.9–1.0 by day 5.

Both MoldVision and human experts achieved low F1 scores on day 1 (range: 18%–32% and 22%–31%, respectively) and day 2 (range: 30%–50% and 28%–46%). By day 3, MoldVision’s F1 scores rose, reaching means of 68%–80% across all four genera, while experts’ scores improved more modestly, to 52%–70%. Notably, MoldVision’s performance exceeded 90% for certain species (e.g., *A. fumigatus *and *Fusarium sp*.) by day 4, while the expert group reached similar levels on day 5 (Fig. 5).

Across all genera, the largest performance gap occurred on days 3 and 4 (mean difference in F1 = 10%–15%), showing that subtle morphologic cues appearing at mid-growth stages were recognized earlier by the MoldVision architecture compared to human observers. By day 5, experts’ scores further converged with MoldVision, demonstrating F1 values approaching 90% for most species. Overall, these data suggest that, while human assessments are dependable as colonies mature, the deep-learning model confers a distinct advantage for earlier detection and classification due to its capacity to detect nuanced colony features at earlier growth stages.

## DISCUSSION

Our study demonstrates a significant advancement in diagnostic culture-based mold identification, highlighting the potential of computer vision models over traditional diagnostic methods. MoldVision exhibited exceptionally good differentiation in days 3–5 growth stages (especially after day 4), achieving AUROC scores that could be considered clinically significant at 98.4% ± 1.3%. The increase in performance in later growth stages aligns with biological differentiation, although it appears to occur more rapidly than expected. Subtle differences in morphological changes may not be picked up easily by human experts but could be detected with a machine learning-based image analysis tool, such as MoldVision. It would be of interest to explore a training approach with more fungal species and integrate more closely related species to determine if the model’s recognition capabilities improve over time. Importantly, this AI tool is not approved according to the rules of the European Union’s *In Vitro* Diagnostics Regulation nor is it authorized for use by the United States Food and Drug Administration, and clearly, our retrospective findings must be prospectively validated and further expanded (30, 31).

Our image data set may serve as an important baseline and proof of concept for continuous training. To the best of our knowledge, (i) there is no publicly available data set that provides standardized images documenting the fungal growth over time and (ii) culture-based morphological criteria such as color, shape, size of the colony, and growth patterns of molds have not been used for machine learning applications. In addition, no comparison to the experience of laboratory technicians has been made up until now. Interestingly, it is well known that *A. fumigatus* and *Penicillium* spp. look highly similar in early growth—a difference which could not be spotted by human readers, but in this study could be reliably distinguished using MoldVision.

Picek et al. (32) developed “FungiVision,” an AI-based fungi species recognition tool for non-medical fungi, specifically identifying environmental fungi based on fruiting bodies. This tool, combined with a citizen-science platform, has substantially increased public engagement and contributed to the accumulation of high-quality fungal species data. The study leverages a novel fine-grained classification data set (33) with important metadata such as habitat and time of observation to enhance classification accuracy. The authors used Vision Transformer architecture and achieved a 46.75% reduction in recognition error compared to earlier methods. The integration of this system within community-driven platforms has streamlined fungal species identification and data collection, fostering a productive collaboration between AI systems and human contributors.

A key distinction between the approach in this study and that of Picek et al. is their implementation of a Vision Transformer, which contrasts with the CNN model used in our study. The selection of a Vision Transformer over a CNN is based on the distinct operational principles of these models. CNNs, as described in this study, function by applying convolutional filters across an image to extract features from localized regions. The process is hierarchical: initial layers detect low-level features, which are progressively aggregated by deeper layers into complex representations. This imparts a strong spatial bias, an assumption that spatially related data are the most relevant. This architecture is well-suited for morphological analysis on standardized media, where consistency and repeating patterns are key.

In contrast, Vision Transformers, as employed by Picek et al., adopt a more global approach. A Transformer partitions an image into patches and uses a self-attention mechanism to compute the contextual relationship of each patch to all others within the image (34). This allows the model to capture long-range dependencies effectively, a major advantage for tasks in highly variable environments where global context is key for correct identification. This, however, also means that Transformers lack the innate spatial bias of CNNs and require substantially larger data sets to accomplish classification without significant overfitting. This is reflected in the Danish data set containing approximately 295,000 images.

The inherent properties of a CNN, therefore, align more closely with the aims of this study. The imaging protocol developed ensures uniform lighting and backgrounds, mitigating the need for the complex context-based modeling at which Transformers excel. Furthermore, the smaller data set available would make a Transformer model highly likely to overfit. The task is more suited to a CNN, as the primary goal is the recognition of specific, localized colony features.

Traditional methods for medical mycology, such as culture-based identification, have remained unexplored in the context of automated species-level identification, though microscopy and molecular techniques have been widely employed. For example, microscopy-based methods, as highlighted in recent studies, focus on the use of AI and CNNs for identifying the fungal genera and species based on microscopic images (23, 35). These approaches often face challenges due to the high visual similarity between fungal species, leading to misidentification.

Studies such as Rahman et al. applied CNNs for fungal species classification, but these methods relied on microscopic features (23). Similarly, Tochigi et al. (36) used AI to distinguish *Aspergillus* spp. from *Mucorales* spp. using histological samples rather than culture-based phenotypes. Although these approaches have provided insights into species-level identification, they have not explored the potential of macroscopic features, which are critical during fungal growth in culture.

Our suggested shift toward using and integrating macroscopic growth characteristics, including colony morphology, as a diagnostic tool is significant, as it complements microscopic techniques and potentially reduces reliance on time-consuming molecular methods (35). To translate MoldVision from a promising research tool into a validated part of the clinical workflow, several practical implementation steps must be considered and researched. A crucial first step would be its integration with existing Laboratory Information Systems to ensure seamless data entry and reporting. This would require developing a user-friendly interface for laboratory technicians, allowing for easy image upload and clear presentation of the model’s prediction along with its confidence score.

New Standard Operating Procedures need to be set up, defining the precise protocol for image acquisition, including lighting, camera angle, and timing, to maintain consistency and accuracy. MoldVision could be implemented as a supplementary diagnostic aid to assist, not replace, human expertise. For instance, its predictions could be used to prioritize cultures for microscopic review or provide a preliminary identification for confirmation by a senior mycologist, potentially shortening the time to final result. This approach would allow the model to be gradually validated in a real-world setting while staff undergo training on its use and limitations. Finally, a robust quality control program, using a panel of well-characterized internal and external quality assessment strains, would be essential to monitor the model’s performance over time and ensure its ongoing reliability for diagnostic support.

Looking to the future, MoldVision could potentially improve diagnostic efficiency in resource-limited settings where advanced microscopy or molecular diagnostics may not be available. From a future perspective, the application of the MoldVision framework warrants consideration. The system’s reliance on low-cost smartphone imaging presents a potential mechanism to mitigate the diagnostic challenges posed by financial constraints and a lack of trained mycologists. This approach could ease the decentralization of preliminary fungal identification, enabling technicians in non-specialized laboratories to generate rapid initial findings.

Furthermore, the platform could be adapted to support a tele-diagnostic model, involving the electronic transmission of images and model-generated classifications from a peripheral laboratory to a central reference facility for expert review. This process would serve to bridge geographic and expertise gaps while also providing a framework for quality assurance and professional development for local personnel. In the long term, the structured collection of identification data across multiple sites might also contribute to valuable epidemiological surveillance of fungal pathogens, an area where data are often sparse.

Our study has several important limitations that should be acknowledged. First, the data set includes only 4,500 fungal images, which is relatively minor compared to standard image libraries typically used for deep learning. The limited number of unique fungal strains increases the risk of overfitting. To address this, we conducted stringent nested cross-validation at the level of plates (and report exclusively test set performance) as well as data augmentation by relying on additional training samples over the course of the longitudinal observation period. This documents the full spectrum of each colony’s morphological development, providing the model with diverse images of varying growth stages and allowing it to learn dynamic growth patterns, which increase informational density per training isolate.

Second, the survey design does not fully and accurately represent the workflow of an experienced lab technician, which could affect the comparison results. The survey presented technicians with only 2D static images (top and bottom views), which do not fully replicate the multi-modal assessment performed in a routine diagnostic workflow. An experienced technician’s workflow is three-dimensional and interactive; they assess colony texture by tilting the plate, observe subtle aerial hyphae, and note characteristics from the side view. Most importantly, macroscopic inspection is nearly always followed by microscopy, which provides definitive cellular-level details for identification. As MoldVision was trained only on macroscopic images, the comparison does not account for the richer data available to a human expert, and its superior performance is confined to this specific, limited task.

Third, the field of machine learning is progressing rapidly. More contemporary models might offer improved performance, greater computational efficiency, or a better ability to capture long-range dependencies in image features. Future research should benchmark MoldVision against newer architectures to ensure modernity.

To enhance and validate the model further, incorporating more diverse data from other research groups is crucial for improving its generalization. Generalization refers to a model’s ability to maintain its performance and accuracy when processing new, unseen data from sources outside of its original training set. A model that performs well on internal data but fails on external data has poor generalization and is of limited clinical or commercial value. A crucial aspect would be to vary the types of smartphones and image angles to create a truly diverse data set. The study used highly standardized light conditions by placing the agar plate in a bespoke metal box. The mode of image acquisition most likely also plays a significant role. Furthermore, incorporating a larger and more taxonomically diverse set of mold isolates would have strengthened the study’s robustness.

This study highlights the substantial potential of computer vision models in mold identification, particularly in later growth stages. By addressing the identified limitations and exploring the suggested future research directions, the field can move closer to deploying these models as reliable diagnostic tools, enhancing the accuracy and efficiency of fungal identification in clinical settings, as well as providing a cost-effective approach for physicians in resource-constrained environments.

## Data Availability

All image data sets of this article have been deposited in the Dryad Digital Repository (https://doi.org/10.5061/dryad.cjsxksnj4). Specifically, the raw PNG stacks (*n* = 4,284) and associated metadata (CSV files listing acquisition parameters) can be accessed at https://datadryad.org/dataset/doi:10.5061/dryad.cjsxksnj4. All custom scripts used for image processing, statistical analysis, and figure generation are available on GitHub at https://github.com/lucius97/MoldVision.

## SUPPLEMENTAL MATERIAL

Supplemental material spectrum.02924-25-s0001.pdf Supplemental Text 1, 2, 3, Figure S1, S2 and Loss Curves.

ASM does not own the copyrights to Supplemental Material that may be linked to, or accessed through, an article. The authors have granted ASM a non-exclusive, world-wide license to publish the Supplemental Material files. Please contact the corresponding author directly for reuse.
