# Supplementary material for: Advancing mold identification in the routine laboratory, performance of smartphone-based imaging and a newly developed convolutional neural network
Source: Microbiol Spectr. 2025 Dec 16;14(2):e02924-25. doi: 10.1128/spectrum.02924-25 (PMC12889110; doi:10.1128/spectrum.02924-25)
Supplement: Supplemental material — Supplemental Text 1, 2, 3, Figure S1, S2 and Loss Curves. [file spectrum.02924-25-s0001.pdf]

SUPPLEMENTAL TEXT 1, SUPPLEMENTAL FIG 1

Brief explanation of Convolutional neural networks

A Convolutional Neural Network (CNN) is a type of deep learning model specifically designed for analyzing structured data such as images (**Supplementary Fig 1**). It is particularly well-suited for image recognition tasks, making it also a powerful tool for microbiology and infectious disease applications, where visual data - such as images of microbial colonies on agar plates, microscopy slides, or histological samples - play a crucial role

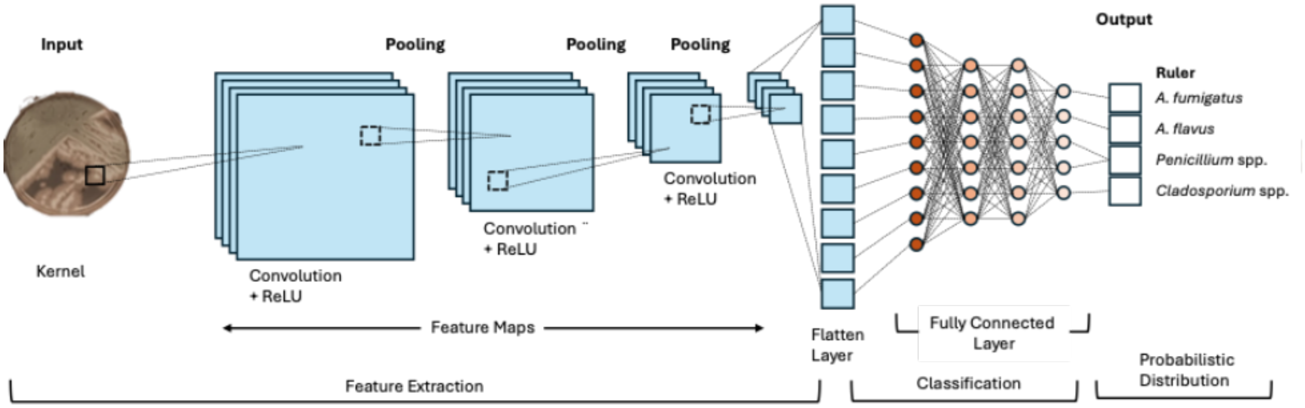

FIGURE 1 Setup of a CNN. The feature extraction in convolutional layers is followed by classification in a fully connected neural network layer, followed by the final probabilistic distribution.

Definition

A CNN is a neural network model that processes images by automatically learning hierarchical patterns and features, such as shapes, textures, and edges, directly from pixel data. These features are progressively combined to detect more complex patterns that enable the network to perform classification tasks, such as identifying different species of microorganisms based on colony morphology.

Structure of CNNs

CNNs are composed of several key layers, each serving a specific function:

1. **Input layer:** This layer takes raw image data, which can be a digital image of microbial growth on an agar plate or a microscopic image of cells.

2. **Convolutional layers:** The core component of a CNN. A convolutional layer applies multiple filters (or kernels) that slide across the image, performing convolution operations. Each filter extracts specific features like edges, shapes, or textures, which are crucial for distinguishing between microbial colonies or pathogens. For example, in detecting mold on agar plates, one filter might highlight the colony's circular shape, while another might detect fine details like surface texture. This is a mathematical operation where a small matrix, called a filter or kernel, slides across an image and multiplies its values with the pixel values at each location. The filter is applied to detect features like edges, textures, or patterns in the image. Convolution helps CNN to focus on important visual characteristics by highlighting regions that may indicate specific features of the mold, such as its shape or texture.

3. **Rectified Linear Unit (ReLU) Activation:** After the convolution operation, the output is passed through the ReLU activation function. ReLU introduces non-linearity to the model by setting any negative pixel values to zero and leaving positive values unchanged. This step is essential because real-world data is often non-linear, and ReLU helps the CNN capture complex patterns in the image data, such as subtle differences between mold species. ReLU also makes the model more efficient by speeding up computations and reducing the risk of vanishing gradients during training.

4. **Pooling layers:** These layers perform down sampling, reducing the spatial dimensions of the feature maps from the convolutional layers while retaining the most important information. Pooling reduces computational complexity and helps the network focus on the most relevant features of the image, such as the distinctive colony morphologies or irregular structures in mold growth.

5. **Fully Connected (Dense) layers:** At the end of the CNN, one or more fully connected layers aggregate all the extracted features and make a final decision about the classification. For example, these layers could output the predicted species of mold or bacteria present in the sample.

6. **Output layer:** The final layer generates a probability distribution over the possible classes (e.g., species of mold or bacteria), allowing the model to make a prediction. The class with the highest probability is typically chosen as the model's prediction.

How CNNs work

There are three critical steps in CNN function.

- **Feature extraction:** CNNs learn to automatically extract key features from the image without the need for manual input or feature engineering. This is particularly useful in microbiology, where subtle variations in colony morphology, texture, or color can be challenging to quantify manually. CNNs excel at detecting these minute details.
- **Training process:** During training, the CNN is fed many labeled images, such as images of different microbial species. The network adjusts its internal parameters (filters, weights, etc.) through a process called backpropagation, using a labeled training dataset. This allows it to learn which visual features are most predictive of the target outcome, such as identifying a specific pathogen based on its colony morphology.
- **Hierarchical learning:** CNNs learn in a hierarchical manner. Initial layers capture low-level features like edges or shapes, while deeper layers capture more complex and abstract features such as colony structures or spatial arrangements of mold filaments, allowing for highly accurate identification and classification.

Example: Image recognition in microbiology

Consider the task of identifying and classifying mold species growing on an agar plate. The raw image is fed into the CNN, which performs the following steps:

1. Convolutional layers extract basic visual features, like the circular shape of the colonies and their surface texture.
2. Pooling layers reduce the size of these feature maps while retaining important information about the mold's structure.

3. ReLU activation helps the network capture complex patterns, such as the unique growth patterns or pigmentation differences.
4. Fully connected layers combine all extracted features and classify the mold into one of five specific genus', such as *Aspergillus spp.* or *Penicillium spp.*

This automated image recognition process streamlines identification and classification, reducing human error and variability. CNNs can be trained to detect multiple microbial species, making them a versatile tool for clinical microbiology and infectious disease diagnostics. They hold promises for applications such as automated bacterial identification, fungal classification, and even pathogen detection in histopathologic images.

Conclusion

CNNs represent a powerful advancement in microbiology and infectious disease research by providing an automated, efficient, and scalable approach to image-based microbial identification. Their ability to learn directly from raw image data, without manual feature extraction, allows for high-accuracy identification of pathogens, aiding in diagnostics and improving clinical outcomes.

SUPPLEMENTAL FIG 2

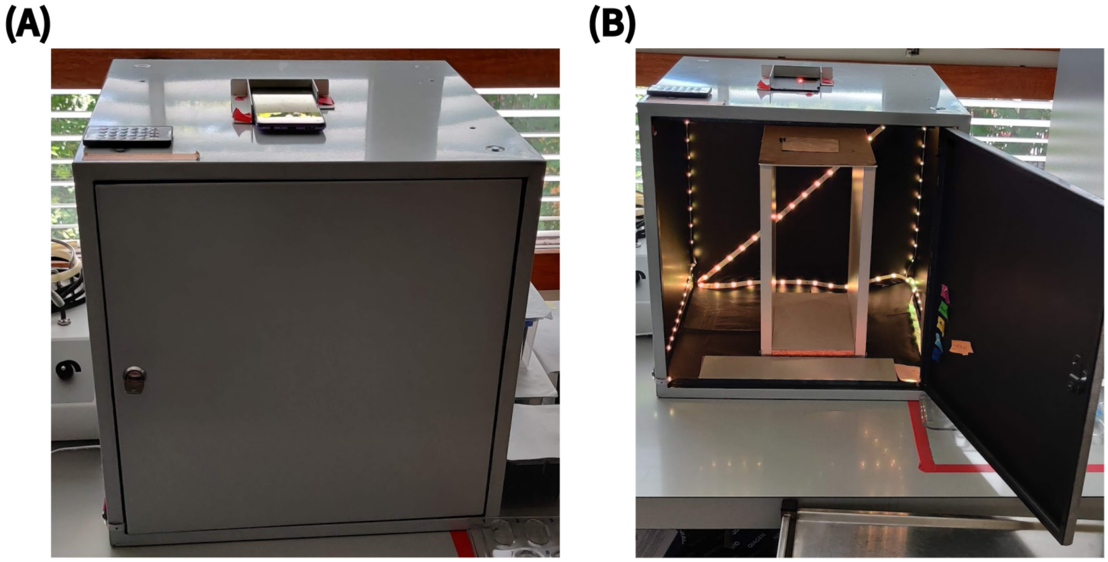

FIGURE 2 (A) Exterior view of the box, showing sealed front panel, latch for sealing and cutout for smartphone. (B) Interior arrangement: user adjustable LED array and specimen platform. Dimensions: approximately 480x480x480mm.

SUPPLEMENTAL TEXT 2

We implemented three variations of the VGG16 model:

1. **Baseline VGG16:** A pre-trained VGG16 model with no modifications, used to process single images independently without linking front and back images.
2. **6-Channel VGG16 (VGG16_6Chan):** This variant modified the top feature extraction layer to accommodate six input channels, integrating both front and back images into a single input tensor.
3. **MoldVision (Twin VGG16):** This model processed front and back images separately through distinct feature extraction and convolutional layers before concatenating their feature maps into a combined feature representation before classification.

The CNNs were developed and implemented using *PyTorch 2.1.0* in *Python 3.12.0* with *CUDA 12.1*. GPUs utilised were NVIDIA V100 Tensor Core GPUs as well as NVIDIA T4 Tensor Core GPUs.

SUPPLEMENTAL TEXT 3

A comprehensive grid search was conducted to determine the optimal LightGBM configuration. The search space comprised:

- a learning rate (η) sweep on a logarithmic scale from 0.05 to 0.001,
- the number of leaves in the range 25-40, and
- a feature fraction interval of 0.80-1.00,

while keeping the boosting algorithm fixed to the standard gradient boosting decision tree (gbdt) mode. Five fold cross validation identified the following setting as optimal: η = 0.005, num_leaves = 31, and feature_fraction = 0.90. These hyper parameters were subsequently adopted for training the final model.

LOSS CURVES

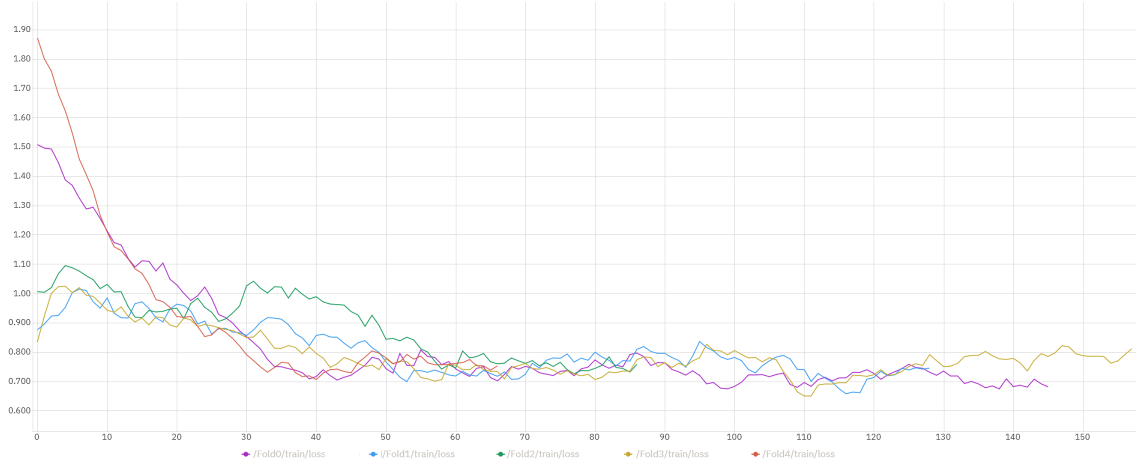

FIGURE 3 Loss Curves for VGG16 Baseline model

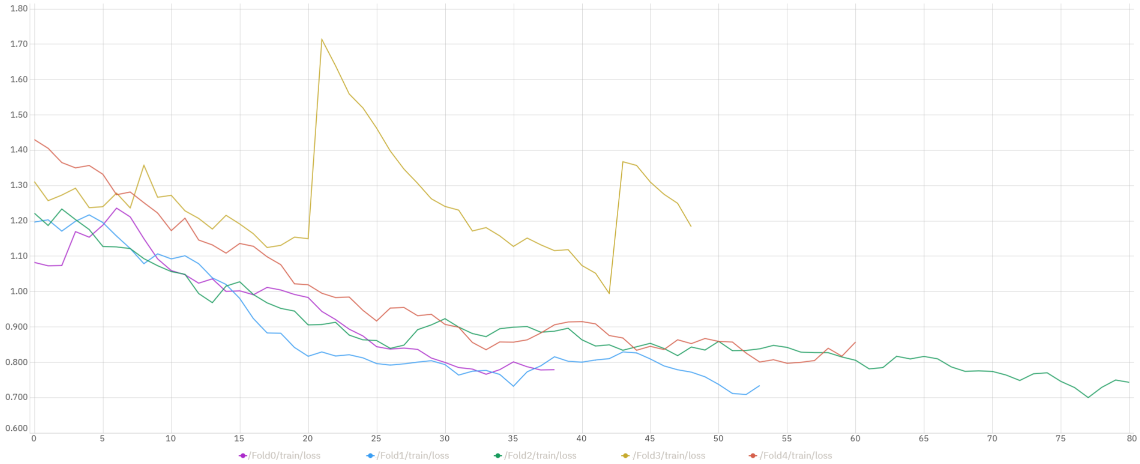

FIGURE 4 Loss Curves for VGG16 6 Channel model

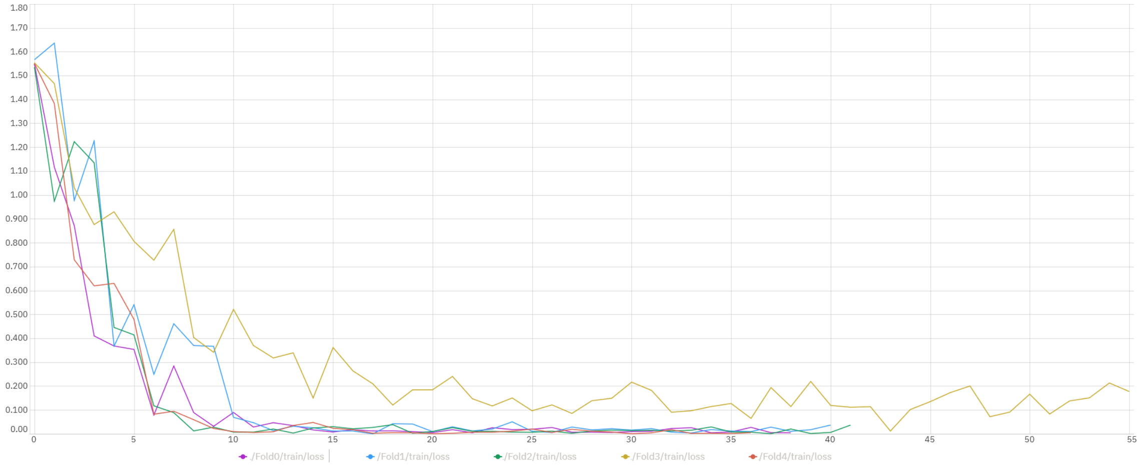

FIGURE 5 Loss Curves for MoldVision model
